# WISER Survivor Trial: Combined Effect of Exercise and Weight Loss Interventions on Adiponectin and Leptin Levels in Breast Cancer Survivors with Overweight or Obesity

**DOI:** 10.3390/nu15153453

**Published:** 2023-08-04

**Authors:** Dan Lin, Kathleen M. Sturgeon, Brett R. Gordon, Justin C. Brown, Dorothy D. Sears, David B. Sarwer, Kathryn H. Schmitz

**Affiliations:** 1 Department of Public Health Sciences, College of Medicine, Pennsylvania State University, Hershey, PA 17033, USA; dul617@psu.edu (D.L.); bgordon1@pennstatehealth.psu.edu (B.R.G.); schmitzk@upmc.edu (K.H.S.); 2 Cancer Metabolism Program, Pennington Biomedical Research Center, Baton Rouge, LA 70808, USA; justin.brown@pbrc.edu; 3 College of Health Solutions, Arizona State University, Phoenix, AZ 85004, USA; dorothy.sears@asu.edu; 4 Department of Medicine, UC San Diego, San Diego, CA 92093, USA; 5 Department of Family Medicine, UC San Diego, San Diego, CA 92093, USA; 6 Moores Cancer Center, US San Diego, San Diego, CA 92093, USA; 7 College of Public Health, Center for Obesity Research and Education, Temple University, Philadelphia, PA 19122, USA; dsarwer@temple.edu; 8 Department of Medicine, University of Pittsburgh, Pittsburgh, PA 15213, USA

**Keywords:** breast neoplasms, obesity, exercise, weight loss, adipokines

## Abstract

Adipocyte dysregulation is one mechanism linking overweight and breast cancer recurrence. Exercise and weight loss are associated with a decreased risk of breast cancer recurrence in breast cancer survivors with overweight or obesity, which may be mediated through reduced leptin levels, increased adiponectin levels, and an elevated adiponectin to leptin (A:L) ratio. The four-arm randomized controlled WISER Survivor trial examined the 12-month intervention effects of exercise, weight loss, and the combination of exercise and weight loss on adipokine levels among breast cancer survivors (*n* = 339) with overweight or obesity. Compared with Control, the Combination of Exercise and Weight Loss decreased leptin levels (−35.9%; 95% CI: −46.8%, −25.0%) and increased A:L ratio (11.6%; 95% CI: 5.6%, 17.6%) but did not change adiponectin levels (4.1%; 95% CI: −3.1%, 11.2%). Compared with Control, Weight Loss Alone decreased leptin levels (−35.6%; 95% CI: −46.6%, −24.5%) and increased A:L ratio (10.6%; 95% CI: 4.7%, 16.5%) but did not change adiponectin levels (0.9%; 95% CI: −6.0%, 7.9%). Compared with Control, Exercise Alone did not change leptin levels, adiponectin levels, or A:L ratio. In analyses that consolidated intervention groups, compared with Control, weight loss of ≥5% decreased leptin levels (*p* trend < 0.01) and increased A:L ratio (*p* trend < 0.01) but did not alter adiponectin levels (*p* trend = 0.53). Weight loss, with or without exercise, was associated with decreased leptin levels in breast cancer survivors with overweight or obesity. Improvements in the adipokine secretion profile (A:L ratio) were primarily driven by a weight loss-induced change in leptin levels.

## 1. Introduction

Breast cancer is the most commonly diagnosed cancer in women in the United States (US), accounting for one-third of new cancer cases among women [1]. The five-year survival rate of breast cancer in the US has increased from 75% in the mid-1970s to 91% in 2018 [2], resulting in an increasing number of breast cancer survivors. The risk of breast cancer recurrence can range from 8% to 38%, depending on nodal status and tumor size at initial diagnosis [3]. An additional risk factor for breast cancer recurrence is excess body weight [4,5,6]. A recent meta-analysis showed that pre- to post-diagnosis weight gain > 10% and obesity (body mass index (BMI) ≥ 30 kg/m^2^) are independently associated with a higher risk of breast cancer recurrence in breast cancer survivors [7].

The mechanisms by which excess weight contributes to breast cancer recurrence are complex and not fully understood. One of the hypothesized mechanisms is abnormal levels of adipokines [8,9]. Adipokines, such as leptin and adiponectin, are proteins secreted by adipocytes [10]. As fat mass increases, adipocytes become dysfunctional, such that the leptin level increases and the adiponectin level decreases [10,11]. Both higher circulating leptin levels and lower circulating adiponectin levels reflect obesity-associated alterations in the adipose tissue adipokinome [12]. Therefore, the adiponectin to leptin (A:L) ratio is suggested as an index to estimate adipose tissue dysfunction [12], and may be useful to assess the net effects of these adipokines on obesity-related diseases, such as breast cancer [13]. Epidemiological evidence supports that a higher leptin level was associated with a higher risk of recurrence in all breast cancer patients [14], and a lower adiponectin level was associated with a higher risk of recurrence in hormone receptor-negative breast cancer patients [15].

Exercise (excess caloric expenditure) and weight loss (caloric restriction) are potential strategies to regulate adipokine levels in breast cancer survivors with overweight or obesity [16,17,18,19,20,21,22,23,24]. However, there is conflicting evidence for the role of increased exercise and caloric restriction on adipokine levels [25]. A previous meta-analysis reported that circulating leptin concentrations in breast cancer survivors were significantly reduced after exercise interventions (weighted mean difference (WMD): −5.7; 95% confidence interval (CI): −11.0, −0.3) but not in combined exercise and weight loss interventions (WMD: −3.0; 95% CI: −7.0, 1.1) [25]. To our knowledge, no previous study has conducted a four-arm randomized controlled trial (RCT) to investigate the independent and combined effects of exercise and weight loss on adipokines in breast cancer patients.

In this study, we evaluated the effects of three interventions compared to a Control arm: Exercise Alone, Weight Loss Alone, and the Combination of Exercise and Weight Loss interventions. We conducted an a priori analysis of secondary outcomes of the Women in Steady Exercise Research (WISER) Survivor trial [26]. We assessed changes in leptin levels, adiponectin levels, and the A:L ratio among breast cancer survivors who had a BMI of ≥25 kg/m^2^. We hypothesized that both Exercise Alone and Weight Loss Alone would decrease leptin levels, increase adiponectin levels, and increase the A:L ratio, with the greatest beneficial change in the Combination of Exercise and Weight Loss group.

## 2. Materials and Methods

### 2.1. Study Design

The WISER Survivor trial was a randomized controlled four-arm trial designed to assess the individual and combined effects of exercise and weight loss interventions over 12 months on lymphedema, body composition, quality of life, and biomarkers for breast cancer recurrence [26]. The study was registered on ClinicalTrials.gov as NCT01515124. The complete study design, recruitment, and main results are described in previous papers [26,27,28,29]. The study was approved by the institutional review board of the University of Pennsylvania, Philadelphia. All participants provided signed informed consent and a written clearance from their physician to participate.

### 2.2. Participants

Breast cancer survivors were eligible for the study if they (1) were 18 to 80 years of age, (2) had stage I-III breast cancer, (3) completed curative treatment at least six months before study enrollment, (4) had a BMI ranging from 25 to 50 kg/m^2^, and (5) had breast cancer-related lymphedema, defined by the Common Toxicity Criteria for Adverse Events, version 4 (National Cancer Institute) [30]. Breast cancer survivors were further excluded if they (1) had medical conditions or medications that would prohibit participation in an exercise program, (2) were unable to walk unaided for more than six minutes, (3) had plans for additional surgery during the study period, (4) engaged in resistance exercise or in aerobic activity of moderate intensity three or more times weekly in the previous 12 months, (5) were using any weight loss medication, (6) lost weight greater than 10% of body weight within the past three months, (7) had self-reported alcohol or substance abuse in the previous 12 months, or (8) had a history of bariatric surgery. In response to recruitment, 2714 respondents expressed interest and were assessed for initial eligibility. Further screening excluded 1491 respondents, and additional reasons detailed in ref. [29] relating to failure to follow up were observed prior to consent and randomization [29].

### 2.3. Randomization

Participants in the WISER survivor trial were randomly assigned to one of the following groups: Control, Exercise Alone, Weight Loss Alone, and the Combination of Exercise and Weight Loss. To account for potential confounders, participants were stratified by age (≤65 and >65 years), baseline BMI (≤37.5 and >37.5 kg/m^2^), radiation (yes and no), number of nodes removed (≤5 and >5), lymphedema severity (grade 1, 2, 3, and 4), and trunk edema only (yes and no). A computerized covariate adaptive procedure was adopted to randomize participants [26]. 

### 2.4. Exercise Intervention

Participants randomized to the Exercise Alone group were asked to engage in two weight training sessions and 180 min of aerobic exercise every week. Weight-adjustable dumbbells were provided for the use of weight training sessions. Participants received in-person, on-site guidance from certified fitness professionals who supervised their weight training and aerobic exercise in the first 6 weeks. The guidance included instructions on resistance training techniques and how to safely increase the duration of aerobic exercise. From weeks 7–52, participants were asked to engage in unsupervised home-based weight training and aerobic exercise using the same routine they followed during the first 6 weeks. Additional monthly supervised in-person sessions were held during this period. Participants were requested to maintain an exercise log to keep track of their exercise adherence. Exercise trainers called participants weekly to offer behavioral counseling and address questions and concerns.

### 2.5. Weight Loss Intervention

Participants randomized to the Weight Loss Alone group were asked to attend weekly group meetings led by a registered dietitian in the first 24 weeks. Meetings aimed to increase adherence to the planned dietary approach through behavioral modification lessons. For the first 20 weeks, participants were asked to follow a meal replacement program (Nutrisystem, Inc.) with a caloric intake restricted to 1200–1500 kcal/day. From weeks 21–24, participants were encouraged to transition to purchasing their own food while following a diet of 1200–1500 kcal/day. From weeks 25–52, participants were asked to increase their caloric intake to 1700–2000 kcal/day with the goal of maintaining the weight they had lost during the first 24 weeks. During this period, adherence to the dietary intervention was monitored through monthly group meetings and weekly calls from the registered dietitian.

### 2.6. Combined Intervention

Participants randomized to the Combination of Exercise and Weight Loss group followed the same exercise protocol as those in the Exercise Alone group for the first 6 weeks. From week 6 onward, they continued the exercise intervention and began receiving the weight loss intervention additionally.

### 2.7. Control Group

Participants randomized to the Control group were asked to maintain their usual level of physical activity and to not engage in any new exercise program, weight training, or supervised weight-loss program. Participants in this group were referred to the American Cancer Society website and their physician if they had questions related to exercise and diet.

### 2.8. Adipokine Measurement

Blood samples from all participants were collected at baseline and 12 months, after a 12 h fast in the morning. After the blood draw, EDTA plasma samples were aliquoted and stored at −80 °C until biomarker measurement. Assay technicians were blinded to group assignment. Plasma leptin concentration was measured using a Meso Scale Discovery immunoassay (Meso Scale Discovery, Cat. #K15164C, Rockville, MD, USA). Plasma concentration of total adiponectin (ng/mL) was measured by an ELISA (ALPCO, Cat. # 80-ADPHU-E01, Salem, NH, USA) and detected on a BioTek EPOCH microplate spectrophotometer (Santa Clara, CA, USA). Intra- and Inter-assay coefficients of variance (CV) were as follows: leptin (5.7%, 12.1%), adiponectin (2.6%, 6.9%).

### 2.9. Body Composition

Body weight was measured by a calibrated scale at baseline and 12 months. Height was measured by a stadiometer at baseline. Body composition was measured with whole-body dual-energy X-ray absorptiometry (DXA; Hologic Inc., Bedford, MA, USA). Detailed descriptions of the study procedures are published elsewhere [26].

### 2.10. Statistical Analysis

Of the 351 women randomized, 339 had plasma samples available for assessment of adipokines at baseline. Those women were considered as the modified intention-to-treat (mITT) population and were included in the analysis (Supplemental Figure S1). Due to breast cancer recurrence or death, 13 women were withdrawn. Of 339 women, 252 had adipokines measured at the study endpoint, week 52. Based on estimates from the Action for Health in Diabetes (Look AHEAD) and the Alberta Physical Activity and Breast Cancer Prevention (ALPHA) trials, this study had 80% statistical power to detect standardized mean difference effect sizes of ≥0.08 for leptin levels using two-sided 0.05-level tests [31,32]. 

Descriptive statistics are presented as a mean (standard deviation (SD)) or median [interquartile range] for continuous variables and *n* (%) for categorical variables. The primary inferential analysis estimated the effect of each of the three intervention groups (Exercise Alone, Weight Loss Alone, and the Combination of Exercise and Weight Loss) compared to the control group using a mixed model for repeated measures using all observed data [33]. The A:L ratio was calculated from log-transformed adiponectin and log-transformed leptin to improve the normality of the data distribution. Treatment effects were calculated as the treatment effect ratio, which determines the percent change in geometric means from baseline to week 52 (e.g., a treatment effect ratio of 0.75 indicates a 25% reduction), with 95% CIs. Regression models included the baseline value of the dependent variable, and randomization stratification factors including age, receipt of radiotherapy, number of lymph nodes resected, lymphedema severity, and body mass index. Group-by-time interaction terms were fixed-effects in the regression models with participant-specific intercepts. Model fit was assessed using a combination of numeric and graphical techniques. All statistical tests were two-sided. Analyses were conducted using Stata/MP v.15.1 (StataCorp LLC, College Station, TX, USA).

## 3. Results

The baseline characteristics of the 339 participants are summarized in Table 1. The study population was 36.6% Black or other minority group. There were no significant differences for age, race, BMI, and cancer stage between groups. Baseline distributions of leptin levels, adiponectin levels, and the A:L ratio were not different between groups. Demographic and clinical characteristics at baseline were not different between the mITT population (*n* = 339) and ITT population (*n* = 351) (Supplemental Table S1). Adherence to aerobic exercise was reported at 140 ± 75 and 156 ± 88 min/wk in the Exercise Alone group and the Combination of Exercise and Weight Loss group, respectively. Attendance at supervised exercise sessions was 83% ± 20% and 86% ± 20% in the Exercise Alone group and the Combination of Exercise and Weight Loss group, respectively.

The baseline and change values of adipokine biomarkers are displayed in Table 2. The intervention effect relative to the Control group is also presented. Compared to the Control, there were no differences in the levels of leptin, adiponectin, and A:L ratio in the Exercise Alone group (−11.3%; 95% CI: −26.4%, 3.8%). Compared to Control, Weight Loss Alone significantly decreased leptin levels (−35.6%; 95% CI: −46.6%, −24.5%) and increased the A:L ratio (10.6%; 95% CI: 4.7%, 16.5%) but did not change adiponectin levels. Similarly, compared to Control, the Combination of Exercise and Weight Loss significantly decreased leptin levels (−35.9%; 95% CI: −46.8%, −25.0%) and increased the A:L ratio (11.6%; 95% CI: 5.6%, 17.6%) but did not change adiponectin levels.

In analyses that consolidated intervention groups, the magnitude of weight loss was strongly associated with changes in leptin levels and the A:L ratio (Table 3). We observed significant linear trends for decreased leptin levels (Δ% (95% CI): weight loss ≥10% vs. control: −60.0 (−66.9, −53.2) vs. 0.0 (reference); *p* trend < 0.001) and an increased A:L ratio (Δ%: (95% CI): weight loss ≥10% vs. control: 25.4 (18.2, 32.7) vs. 0.0 (reference); *p* trend < 0.001) with greater weight loss. Weight loss did not change adiponectin levels significantly (*p* trend = 0.53).

Changes in adipokines in relationship to other previously reported changes in outcomes related to body composition [28], metabolism [34], and inflammation [35] were further examined (Supplemental Table S2). Exploratory observations indicated that changes in leptin and adiponectin levels were differently responsive to changes in the visceral fat area (VFA) and serum amyloid A (SAA). SAA is a cytokine-like protein linking increased adipose tissue mass and low-grade inflammation in obesity. Changes in leptin levels were positively correlated with changes in the VFA (r = 0.35; *p* < 0.001) and SAA (r = 0.24, *p* < 0.001), whereas changes in adiponectin levels were not correlated with changes in these two biomarkers.

## 4. Discussion

We investigated the changes in circulating adipokine levels after 52 weeks of Exercise Alone, Weight Loss Alone, or the Combination of Exercise and Weight Loss interventions among breast cancer survivors who had a BMI of ≥25 kg/m^2^. Weight Loss Alone and the Combination of Exercise and Weight Loss decreased leptin levels and increased the A:L ratio. There were no significant changes in adiponectin levels. Exercise Alone was not associated with changes in leptin levels, adiponectin levels, or the A:L ratio. A dose–response association between weight loss and leptin levels, as well as the A:L ratio, was observed. Greater weight loss was associated with lower leptin levels and a higher A:L ratio. The results of this study suggest that the reduction of pro-inflammatory leptin levels was highly responsive to weight loss, while increases in anti-inflammatory adiponectin levels were not. 

Decreased leptin levels following the weight loss intervention, either alone or in combination with exercise, in our study, was consistent with observations from other groups [16,17,36,37]. In addition, similar to our finding, an increased A:L ratio was observed following a six-month weight loss intervention with a restriction of 1200–1500 kcal/day diet in breast cancer survivors who were either overweight or had obesity [16]. Our observations add to the literature by suggesting that weight loss alone or in combination with exercise is more effective than exercise alone in the favorable modulation of leptin levels and the A:L ratio.

Leptin can stimulate the growth and proliferation of breast cancer cells by binding to its receptors on these cells [38]. This can lead to the activation of signaling pathways that promote cell growth and survival [38]. Leptin supports metastatic progression and tumor recurrence by increasing invasion and migration potential as well as stem-like characteristics in breast cancer cells [39]. In particular, an elevated leptin level due to dysfunctional adipose tissue may induce activation of dormant breast tumor cells [40]. Higher leptin levels are associated with an increased risk of breast cancer recurrence and worse overall survival in breast cancer patients [14]. Therefore, reducing leptin levels through weight loss, with or without exercise, may help to reduce the risk of recurrence and improve outcomes for breast cancer survivors.

Similar to our observations, other studies did not observe changes in adiponectin levels in breast cancer survivors who experience weight loss [17,21,22,36,37,41]. Our results are further supported by a recent meta-analysis demonstrating that the combination of exercise and diet-induced weight loss had a non-significant change in adiponectin levels [25]. Only one single-arm study reported an increase in adiponectin levels following a six-month weight loss alone intervention [16]. Given the lack of overall response of this adipokine to changes in body weight, future RCTs may need to identify and characterize individuals as adiponectin “responders” and “non-responders” to energetic interventions.

A 52-week exercise intervention of progressive weightlifting and aerobic exercise did not change leptin levels, adiponectin levels, or the A:L ratio. Previous observations for the effect of exercise on leptin levels in breast cancer survivors were contradictory [18,19,23,24]. Two studies with small sample sizes (<30 participants) reported that an aerobic and resistance combination exercise intervention decreased leptin levels [18,23]. Two studies with larger sample sizes (>100 participants), consistent with our finding, observed no significant change in leptin levels [19,24], indicating that the positive effect of exercise on leptin levels in breast cancer survivors is still unclear. Similarly, the effect of exercise on adiponectin levels in breast cancer survivors appears to be inconsistent among studies. The non-significant change in adiponectin levels following exercise intervention in our study was in line with two previous meta-analyses [20,25]. Yet, increased adiponectin levels following an exercise intervention were observed in one small RCT (<20 participants) [18].

We observed dose–response associations between weight loss and leptin levels and the A:L ratio. In comparison to the control group, and independent of the intervention arm, weight loss (5.0–9.9% of baseline weight or ≥10% of baseline weight) was associated with lower leptin levels and a higher A:L ratio. These findings were in line with the CHOICE study, which reported a significant decrease in leptin levels with >15% of baseline weight loss [37]. Clinically meaningful weight loss (associated with decreased risk of type 2 diabetes, cancer, and adverse cardiovascular events [42,43,44]) occurs at 5% or more of baseline weight loss in patients with overweight or obesity [45]. Thus, the observed magnitude of weight loss in the WISER Survivor trial that was associated with leptin level reduction and A:L ratio increase could also impact the risk of other chronic diseases [46,47].

Given the overall strong effects of weight loss on leptin levels, but the null effects on adiponectin levels, we explored adipokine relationships with other metrics of weight loss (body composition changes and changes in biomarkers related to chronic disease). A strong correlation between the change in leptin levels and fat mass was consistent with observations from other human and mouse models [48,49]. Previous research has also indicated that the leptin level was highly correlated with adipocyte size [50]. We observed that adipokine relationships with the VFA and SAA differed. Differences were noted for associations between changes in adipokines and changes in the VFA. Specifically, decreases in the VFA exhibited a positive correlation with decreases in leptin levels, while no such correlation was observed with adiponectin levels. Changes in the VFA had a particularly weak relationship with changes in the adiponectin level, compared to changes in the subcutaneous fat area and total fat mass. An in vitro study showed that adiponectin gene expression is lower in visceral adipose tissue than in subcutaneous adipose tissue [51], suggesting subcutaneous adipose tissue may contribute to circulating adiponectin levels to a greater extent. We also observed that changes in adiponectin levels were not consistently associated with changes in SAA levels, as changes in leptin levels were. A study found that serum concentrations of leptin and SAA were interrelated independent of body fat mass in an obese population [52]. However, the relationship between SAA and adiponectin levels in overweight or obese populations is not clear yet. Our observations suggest future exploration of the biology of the VFA and SAA in relationship to adipokines. Understanding this mechanism may provide insights into the underlying causes and potential interventions (e.g., weight loss) for various health conditions associated with obesity.

The WISER Survivor trial was accomplished under the umbrella of the Transdisciplinary Research on Energetics and Cancer (TREC) initiative [53]. This initiative focused on interrelated projects that spanned the continuum of translational research [26]. As another part of the TREC initiative, a preclinical mouse model paralleled the WISER Survivor trial and investigated the effect of obesity on breast cancer recurrence and biomarkers of breast cancer in a genetically engineered mouse model of breast cancer recurrence [54]. By comparing data from animals to humans, the TREC initiative aimed to answer questions regarding adiposity and cancer survivorship through a transdisciplinary approach.

The animal study in parallel with the WISER Survivor trial demonstrated results similar to observations in humans: obese mice exhibited an increased incidence and a faster rate of mammary tumor recurrence compared to lean mice [54]. Further, obese mice had higher circulating levels of leptin and lower levels of adiponectin compared to lean mice, despite both groups being fed high-fat diets [54]. These findings suggest that, independent of diet quality, excess adiposity and subsequent dysregulation of adipokines increase the risk of breast cancer recurrence. Our study included breast cancer survivors who were overweight or obese at baseline and observed that weight loss was associated with decreased leptin levels and an increased A:L ratio. While the animal study did not have a weight loss intervention, the observed positive association between obesity, leptin levels, and breast cancer recurrence provided a biological rationale for conducting weight loss interventions on breast cancer survivors with overweight or obesity.

Several strengths of the study are worth highlighting. First, 124 of the 339 participants (36.6%) were of a minority race. This diverse representation enhances the generalizability of the findings. Second, we were able to assess the independent and combined effects of two lifestyle interventions in a single trial. The four-armed design allowed for a time- and cost-efficient comparison of two energetic interventions. Third, the intervention in our trial was deployed using a combination of in-person and home-based methods. The use of a home-based exercise intervention was successfully adopted from previous research and reduced the barriers to participants for gym-based programs [26]. The meal replacement program used for weight loss intervention was commercially available and accessible across the US.

The study has some limitations. First, the WISER Survivor trial was designed to evaluate the effect of interventions on lymphedema outcomes in breast cancer survivors. As such, exercise and weight loss interventions were prescribed specifically for lymphedema outcomes. Changes in adipokines that might be prognostic for breast cancer outcomes may require different exercise and weight loss prescriptions. Second, we measured total adiponectin levels rather than high-molecular-weight adiponectin levels. High-molecular-weight adiponectin is a more bioactive form of adiponectin and may be considered as a better predictor of metabolic dysfunction [55]. Third, our four-arm study design, which included Exercise Alone and Weight Loss Alone arms, as well as the Combination of Exercise and Weight Loss arm, was unable to test the interaction effect of exercise and weight loss on adipokines levels. To investigate whether the effectiveness of one intervention is dependent on the level of the other, future research should consider a 2 × 2 factorial design. Fourth, we did not collect information on co-morbidities from women in the study. Thus, we cannot speculate on how adipokines may be associated with, or change in, other co-occurring diseases in breast cancer survivors.

## 5. Conclusions

In conclusion, analysis suggests that weight loss with or without exercise is effective for decreasing leptin levels and increasing the A:L ratio in breast cancer survivors with overweight or obesity. The decrease in leptin levels represents a favorable modulation because of its association with a lower risk of breast cancer recurrence. Encouraging breast cancer survivors with excess weight to target at minimum a five percent weight loss may decrease the risk of breast cancer recurrence.

## Figures and Tables

**Table 1:** Baseline characteristics by randomized group.

Characteristic	Control (*n* = 88)	Exercise (*n* = 84)	Weight Loss (*n* = 84)	Exercise and Weight Loss (*n* = 83)
Age, y	59.0 (8.6)	59.2 (8.2)	59.3 (9.2)	60.0 (9.1)
Race, *n* (%)				
White	64 (72.7%)	49 (58.3%)	52 (61.9%)	50 (60.2%)
Black	22 (25.0%)	34 (40.5%)	29 (34.5%)	28 (33.7%)
Other	2 (2.3%)	1 (1.2%)	3 (3.6%)	5 (6.1%)
Body mass index, kg/m^2^	34.0 (5.7)	34.2 (6.2)	33.8 (5.5)	34.0 (6.1)
Cancer stage, *n* (%)				
Ductal carcinoma in situ	10 (11.4%)	6 (7.1%)	5 (6.0%)	3 (3.6%)
I	18 (20.4%)	24 (28.6%)	17 (20.2%)	14 (16.9%)
II	22 (25.0%)	22 (26.2%)	29 (23.5%)	26 (31.3%)
III	16 (18.2%)	13 (15.5%)	19 (22.6%)	20 (24.1%)
Unknown	22 (25.0%)	19 (22.6%)	14 (16.7%)	20 (24.1%)
Leptin, ng/mL	58.9 [30.9, 83.6]	53.1 [31.7, 80.1]	54.0 [35.6, 77.4]	57.9 [31.9, 77.0]
Adiponectin, μg/mL	6.5 [5.1, 9.6]	6.8 [5.1, 9.0]	6.5 [4.4, 8.3]	7.1 [5.1, 10.8]
A:L ratio ^1^	0.49 [0.38, 0.59]	0.48 [0.39, 0.60]	0.47 [0.36, 0.56]	0.50 [0.38, 0.63]

Values are mean (standard deviation), median [interquartile range], or *n* (%).^1^ The A:L ratio was calculated from log-transformed adiponectin and log-transformed leptin to improve the normality of the data distribution.

**Table 2:** Change in adipokine biomarker endpoints by randomized group.

Adipokine Endpoint	Randomized Group	Baseline Geometric Mean (SD)	Geometric Mean Change (SE)	Intervention Main Effect, Treatment Ratio (95% CI)	Percent Difference between Groups (95% CI)
Leptin, ng/mL	Control	3.91 (0.72)	0.03 (0.06)	1.00 (Reference)	0.00 (Reference)
	Exercise	3.86 (0.72)	−0.09 (0.06)	0.89 (0.74, 1.04)	−11.3 (−26.4, 3.8)
	Weight Loss	3.88 (0.66)	−0.41 (0.06) ^1^	0.64 (0.53, 0.75) ^2,3^	−35.6 (−46.6, −24.5) ^2,3^
	Exercise and Weight Loss	3.89 (0.69)	−0.42 (0.06) ^1^	0.64 (0.53, 0.75) ^2,3^	−35.9 (−46.8, −25.0) ^2,3^
Adiponectin, μg/mL	Control	1.91 (0.43)	0.05 (0.02)	1.00 (Reference)	0.00 (Reference)
	Exercise	1.89 (0.49)	−0.01 (0.02)	0.95 (0.88, 1.01)	−5.5 (−11.9, 1.0)
	Weight Loss	1.80 (0.45)	0.06 (0.02) ^1^	1.01 (0.94, 1.08)	0.9 (−6.0, 7.9)
	Exercise and Weight Loss	1.90 (0.47)	0.09 (0.02) ^1^	1.04 (0.97, 1.11)	4.1 (−3.1, 11.2)
A:L ratio	Control	0.51 (0.17)	0.01 (0.02)	1.00 (Reference)	0.00 (Reference)
	Exercise	0.52 (0.19)	0.04 (0.02)	1.03 (0.98, 1.09)	3.5 (−2.0, 9.0)
	Weight Loss	0.49 (0.19)	0.10 (0.02) ^1^	1.11 (1.05, 1.16) ^2,3^	10.6 (4.7, 16.5) ^2,3^
	Exercise and Weight Loss	0.53 (0.20)	0.11 (0.02) ^1^	1.12 (1.06, 1.18) ^2,3^	11.6 (5.6, 17.6) ^2,3^

Data were analyzed using a mixed model for repeated measures using observed data. Models are adjusted for the baseline value of the dependent variable, and randomization stratification factors including age, receipt of radiotherapy, number of lymph nodes resected, lymphedema severity, and body mass index. ^1^ *p* < 0.05 (two-sided) compared to baseline (within group). ^2^ *p* < 0.05 (two-sided) compared to control. ^3^ *p* < 0.05 (two-sided) compared to exercise. *p* values are not adjusted for multiplicity.

**Table 3:** Change in adipokine biomarker outcomes by the magnitude of weight loss.

Adipokine Endpoint	Group	Baseline Geometric Mean (SD)	Geometric Mean Change (SE)	Intervention Main Effect, Treatment Ratio (95% CI)	Percent Difference between Groups (95% CI)
Leptin, ng/mL	Control	3.87 (0.75)	0.03 (0.06)	1.00 (Reference)	0.00 (Reference)
	<5.0%	3.86 (0.70)	−0.02 (0.05)	0.96 (0.81, 1.10)	−4.5 (−18.6, 9.7)
	5.0–9.9%	3.73 (0.82)	−0.29 (0.08) ^1^	0.73 (0.59, 0.87) ^2^	−27.1 (−41.5, −12.6) ^2^
	≥10%	3.95 (0.53)	−0.89 (0.06) ^1^	0.40 (0.33, 0.47) ^2^	−60.0 (−66.9, −53.2) ^2^
					*p* trend < 0.0001
Adiponectin, μg/mL	Control	1.94 (0.43)	0.05 (0.03)	1.00 (Reference)	0.00 (Reference)
	<5.0%	1.93 (0.48)	0.03 (0.02)	0.98 (0.90, 1.05)	−2.2 (−9.7, 5.3)
	5.0–9.9%	1.85 (0.43)	0.02 (0.04)	0.97 (0.87, 1.06)	−3.5 (−13.3, 6.4)
	≥10%	2.00 (0.46)	0.12 (0.03)^1^	1.07 (0.97, 1.16)	6.7 (−2.9, 16.3)
					*p* trend = 0.53
A:L ratio	Control	0.52 (0.17)	0.01 (0.02)	1.00 (Reference)	0.00 (Reference)
	<5.0%	0.52 (0.18)	0.01 (0.01)	1.01 (0.96, 1.06)	1.0 (−4.0, 5.9)
	5.0–9.9%	0.53 (0.24)	0.09 (0.03) ^1^	1.09 (1.02, 1.16) ^2^	9.1 (2.0, 16.2) ^2^
	≥10%	0.52 (0.16)	0.23 (0.02) ^1^	1.25 (1.18, 1.33) ^2^	25.4 (18.2, 32.7) ^2^
					*p* trend < 0.0001

Data were analyzed using a mixed model for repeated measures using observed data. Models are adjusted for the baseline value of the dependent variable and randomization stratification factors including age, receipt of radiotherapy, number of lymph nodes resected, lymphedema severity, and body mass index. ^1^ *p* < 0.05 (two-sided) compared to baseline (within group). ^2^ *p* < 0.05 (two-sided) compared to control. *p* values are not adjusted for multiplicity.

## Data Availability

All data used in this study can be made publicly available upon reasonable request.

## Supplementary Materials

The following supporting information can be downloaded at: https://www.mdpi.com/article/10.3390/nu15153453/s1, Figure S1: Flow of participants by randomized group and definition of the intention-to-treat (ITT) and modified intention-to-treat (mITT) populations; Table S1: Baseline characteristics between the intention-to-treat (ITT) and modified-intention-to-treat (mITT) populations; Table S2: Correlation of log-transformed change in adipokine biomarkers by change in body composition and biomarkers of insulin resistance, inflammation, and sex steroid hormones.
