# Supplementary material for: WISER Survivor Trial: Combined Effect of Exercise and Weight Loss Interventions on Adiponectin and Leptin Levels in Breast Cancer Survivors with Overweight or Obesity
Source: Nutrients. 2023 Aug 4;15(15):3453. doi: 10.3390/nu15153453 (PMC10421485; doi:10.3390/nu15153453)
Supplement: Supplementary file 1 [file nutrients-15-03453-s001.zip › nutrients-2514288-supplementary.pdf]

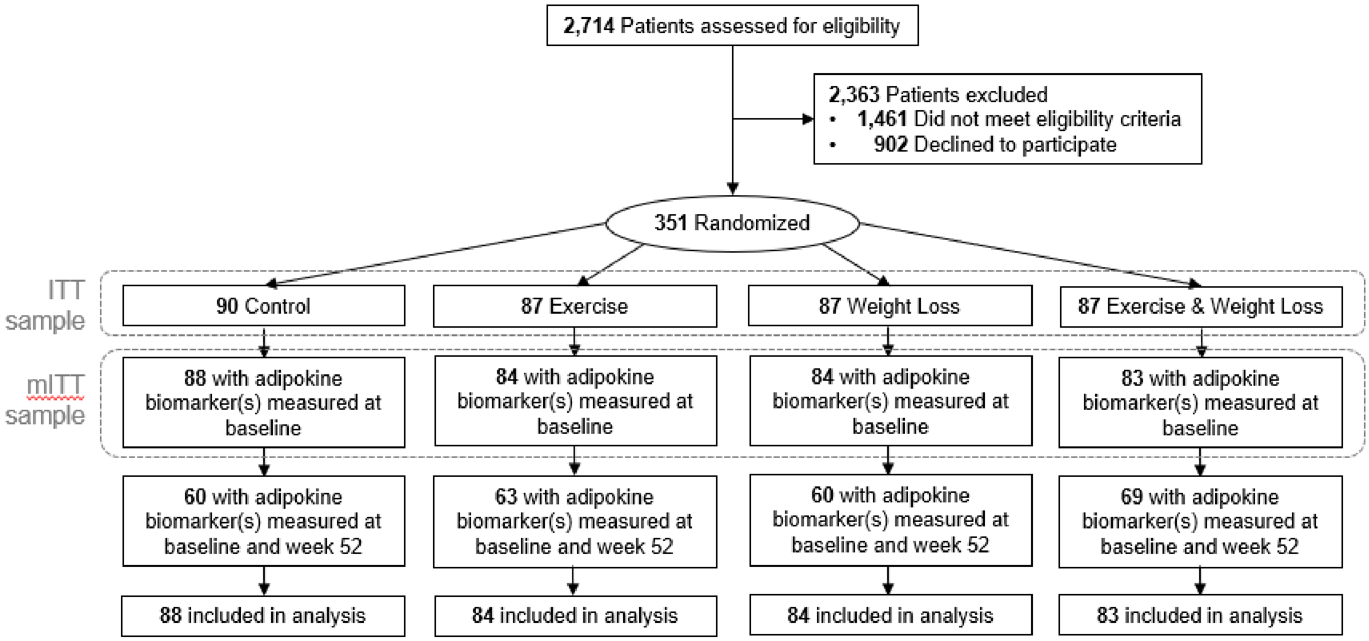

Figure S1. Flow of participants by randomized group and definition of the intention to teat (ITT) and modified intention-to-treat (mITT) populations.

Table S1. Baseline characteristics between the intention-to-treat (ITT) and modified-intention-to-treat (mITT) populations.

Characteristic	ITT (n=351)	mITT (n=339)
Age, y	59.4 (8.7)	59.4 (8.7)
Race, n (%)		
White	218 (62.1%)	215 (63.4%)
Black	122 (34.8%)	113 (33.3%)
Other	11 (3.9%)	11 (3.3%)
Body mass index, kg/m^2	34.0 (5.9)	34.0 (5.9)
25.0–29.9 kg/m^2	108 (30.8%)	104 (30.7%)
30.0–34.9 kg/m^2	102 (29.1%)	98 (28.9%)
≥35.0 kg/m^2	141 (40.1%)	137 (40.4%)
Cancer stage, n (%)		
Ductal carcinoma in situ	24 (6.8%)	24 (7.1%)
I	74 (21.1%)	73 (21.5%)
II	104 (29.6%)	99 (29.2%)
III	70 (19.4%)	68 (20.1%)
Unknown	79 (23.1%)	75 (22.1%)
No. of nodes removed, n	12.5 (9.2)	12.5 (9.2)
Cancer treatments, n (%)		
Chemotherapy	289 (82.3%)	277 (81.7%)
Radiotherapy	288 (82.1%)	278 (82.0%)
Tamoxifen	36 (10.3%)	35 (10.3%)
Aromatase inhibitor	102 (29.1%)	101 (29.8%)
Time since cancer diagnosis, mo.	92.0 (63.4)	92.7 (63.7)

Values are mean (standard deviation) or n (%).

Table S2. Correlation of log-transformed change in adipokine biomarkers by change in body composition and biomarkers of insulin resistance, inflammation, and sex steroid hormones.

	**Δ Leptin**	**Δ Adiponectin**	**Δ Adiponectin: Leptin Ratio**
Δ Body weight	*r*=0.65; *P*<0.001	*r*=–0.21; *P*=0.001	*r*=–0.53; *P*<0.001
Δ Fat mass	*r*=0.63; *P*<0.001	*r*=–0.19; *P*=0.004	*r*=–0.50; *P*<0.001
Δ Visceral fat area	*r*=0.35; *P*<0.001	*r*=–0.06; *P*=0.33	*r*=–0.24; *P*<0.001
Δ Subcutaneous fat area	*r*=0.48; *P*<0.001	*r*=–0.15; *P*=0.020	*r*=–0.44; *P*<0.001
Δ Lean mass	*r*=0.39; *P*<0.001	*r*=–0.23; *P*<0.001	*r*=–0.34; *P*<0.001
Δ Appendicular lean mass	*r*=0.32; *P*<0.001	*r*=–0.18; *P*=0.006	*r*=–0.25; *P*<0.001
Δ Insulin	*r*=0.04; *P*=0.53	*r*=–0.08; *P*=0.25	*r*=0.04; *P*=0.56
Δ Glucose	*r*=0.15; *P*=0.020	*r*=–0.22; *P*=0.001	*r*=–0.18; *P*=0.006
Δ C-peptide	*r*=0.51; *P*<0.001	*r*=–0.19; *P*=0.004	*r*=–0.40; *P*<0.001
Δ C-reactive protein	*r*=0.26; *P*<0.001	*r*=–0.20; *P*=0.003	*r*=–0.24; *P*<0.001
Δ Serum amyloid A	*r*=0.24; *P*<0.001	*r*=–0.11; *P*=0.11	*r*=–0.21; *P*=0.002
Δ ICAM-1	*r*=0.03; *P*=0.69	*r*=–0.14; *P*=0.039	*r*=–0.09; *P*=0.20
Δ VCAM-1	*r*=–0.08; *P*=0.22	*r*=–0.05; *P*=0.50	*r*=0.04; *P*=0.52
Δ Estradiol	*r*=0.12; *P*=0.089	*r*=0.03; *P*=0.61	*r*=–0.04; *P*=0.58
Δ SHBG	*r*=–0.37; *P*<0.001	*r*=0.27; *P*<0.001	*r*=0.39; *P*<0.001
Δ Testosterone	*r*=0.23; *P*=0.004	*r*=–0.14; *P*=0.067	*r*=–0.22; *P*=0.005

HOMA-IR, homeostatic model assessment of insulin resistance; ICAM-1, intercellular adhesion molecule 1; vascular cell adhesion molecule 1; SHBG, sex hormone binding globulin
